# Colorful Niches of Phytoplankton Shaped by the Spatial Connectivity in a Large River Ecosystem: A Riverscape Perspective

**DOI:** 10.1371/journal.pone.0035891

**Published:** 2012-04-30

**Authors:** Jean-Jacques Frenette, Philippe Massicotte, Jean-François Lapierre

**Affiliations:** 1 Département de Chimie-Biologie, Université du Québec à Trois-Rivières, Trois-Rivières, Québec, Canada; 2 Département des sciences biologiques, Université du Québec à Montréal, Montréal, Québec, Canada; US Dept. of Agriculture – Agricultural Research Service (USDA-ARS), United States of America

## Abstract

Large rivers represent a significant component of inland waters and are considered sentinels and integrators of terrestrial and atmospheric processes. They represent hotspots for the transport and processing of organic and inorganic material from the surrounding landscape, which ultimately impacts the bio-optical properties and food webs of the rivers. In large rivers, hydraulic connectivity operates as a major forcing variable to structure the functioning of the riverscape, and–despite increasing interest in large-river studies–riverscape structural properties, such as the underwater spectral regime, and their impact on autotrophic ecological processes remain poorly studied. Here we used the St. Lawrence River to identify the mechanisms structuring the underwater spectral environment and their consequences on pico- and nanophytoplankton communities, which are good biological tracers of environmental changes. Our results, obtained from a 450 km sampling transect, demonstrate that tributaries exert a profound impact on the receiving river’s photosynthetic potential. This occurs mainly through injection of chromophoric dissolved organic matter (CDOM) and non-algal material (tripton). CDOM and tripton in the water column selectively absorbed wavelengths in a gradient from blue to red, and the resulting underwater light climate was in turn a strong driver of the phytoplankton community structure (prokaryote/eukaryote relative and absolute abundances) at scales of many kilometers from the tributary confluence. Our results conclusively demonstrate the proximal impact of watershed properties on underwater spectral composition in a highly dynamic river environment characterized by unique structuring properties such as high directional connectivity, numerous sources and forms of carbon, and a rapidly varying hydrodynamic regime. We surmise that the underwater spectral composition represents a key integrating and structural property of large, heterogeneous river ecosystems and a promising tool to study autotrophic functional properties. It confirms the usefulness of using the riverscape approach to study large-river ecosystems and initiate comparison along latitudinal gradients.

## Introduction

Inland waters are sentinels and integrators of terrestrial and atmospheric processes [1]. They represent hotspots for the transport and processing of organic and inorganic material drained from the surrounding terrestrial landscape. In terms of magnitude, the amount of carbon that is exported from terrestrial to aquatic ecosystems is comparable to the terrestrial carbon sink [2]. Lakes, reservoirs, rivers and wetlands have been shown to return about half of that carbon to the atmosphere, in the form of carbon dioxide, before it reaches the oceans [3], and rivers and streams have recently been identified as major contributors to this carbon loss [4], [5]. This reflects the tremendous amount of material that travels from land to oceans via riverine ecosystems and suggests that this material shapes carbon-cycling dynamics in the receiving systems in several ways.

In addition to representing a direct substrate for mineralization, this terrestrial material, which includes dissolved organic matter (DOM), nutrients, and organic and inorganic sediments, also affects the flow of carbon in aquatic foodwebs. Terrestrial organic carbon may be passed directly to primary consumers through heterotrophy [6] and terrestrial nutrients may support aquatic primary productivity [7]. Through their impact on the underwater light climate, the combined effect of these terrestrial inputs also indirectly shapes primary producer communities (e.g. [8]). Therefore, bio-optical properties of water bodies and associated color spectra have been recognized as key elements that describe the structure and spatial heterogeneity of many aquatic ecosystems [9]. Kirk [10] further linked water optics to water quality through a concept called “optical water quality”, which has been mostly applied to standing water, including lakes or oligotrophic oceanic waters.

The mechanisms driving the bio-optical properties of water, however, differ fundamentally between inland and marine systems, so patterns that are well-established in a system may not be exportable to another. For instance, in marine systems, chlorophyll *a* (Chl*a*), a general indicator of the phytoplankton biomass, is one of the main factors that determines the optical properties of the surface water [11]. Conversely, since dissolved organic matter (DOM) imported from the terrestrial landscape tends to be highly colored or chromophoric (CDOM), it has a high impact on the underwater light climate in inland water systems, more particularly at the level of underwater optical properties such as ultraviolet (UV) and infrared radiation or color attenuation [1], [9], [12]. Light attenuation in inland waters is thus strongly influenced by watershed characteristics, rather than by biological components per se.

Spatial connectivity, i.e. transfer of matter and energy between habitats, appears to be an important driver of DOM dynamics, as a large fraction of environmental heterogeneity is attributable to the upstream-downstream gradient and the aquatic-terrestrial exchanges via imports of different types of DOM from the tributaries [13]. However, widespread regional changes in CDOM concentrations in inland waters remain poorly studied in general [14] and, more specifically, at the level of streams and rivers, where the impact of watershed characteristics on the underwater light spectrum is largely unknown. Part of this lack of study is attributable to the difficulty of measuring light characteristics in stream environments because of the small size and shallow depth of streams [15], and to the challenges of conducting multiscale studies in large-river ecosystems [16].

The St. Lawrence River (SLR) is a representative model of a large-river ecosystem, integrating a wide variety of landscape conditions, thus facilitating such multiscale studies. It is the second-largest river in North America in terms of discharge [17] and, based on the 167 largest world rivers, is 19th and 20th, respectively, in terms of drainage area and average discharge rate (World Resources Institute: http://www.wri.org) (see Fig. S1 and Text S1). The SLR forms a complex system composed of a mosaic of heterogeneous zones such as fluvial lakes, connecting reaches and wetlands, which interacts with inflowing tributaries to produce strong longitudinal and lateral connectivity between aquatic and terrestrial environments. Large rivers such as the SLR are characterized by physical continuities and discontinuities that operate at various spatial scales along both the longitudinal and lateral axes. Terrestrial-aquatic exchanges between the tributaries and the landscape, superimposed over the river’s hydrology, bathymetry, climate characteristics and drainage network [18], [19] exert a significant impact on the physical diversity of the receiving river [20], [21]. For instance, water intrusions from tributaries become distinct water masses that flow down the river and contribute to spatial and temporal heterogeneity in optical characteristics and transport times [20], [22], [23]. Through the amount and type of material transferred between habitats, this asymmetric and directional connectivity among the river’s habitats further contributes to structuring the river’s attributes. The resulting heterogeneity further defines the concept of “riverscape” (*sensu* [24]), which deals with the influence of spatial patterns on riverine ecological processes.

All these discontinuities interact and provide a gradient of physical heterogeneity, which is likely to affect the biocomplexity of the riverscape. However, empirical evidence demonstrating the ecological importance of physical heterogeneity on biological components is limited for large-river ecosystems. The first such limitation is the lack of appropriate integration of physical processes [19] and related biological components over spatial scales representative of large rivers. A notable exception is the study conducted by Hoeinghaus et al. [25], who compared the energy flow from autotrophs to consumers in four different multiscale landscape types based on hydrologic gradients in the large Paraná River, which is in the tropical latitudes. However, they could not identify key structural and integrating riverscape properties that both acted as proximal factors of energy flow and would explain the disparity in their results. They concluded with the need to incorporate landscape-scale hydrologic variables into large-river studies. Available studies in the SLR (e.g. [26]–[29]) provide limited-scale descriptions of environmental and biological responses, which do not allow for a global understanding of the prevailing changes occurring along the riverscape ([24]) and the resulting consequences for the biota. As a result, they bring little evidence for the underlying mechanisms that control the spatio-temporal variability from the outlet of the Great Lakes to the marine intrusion, 450 kilometers downstream at the estuarine transition zone (ETZ).

Thus, we surmise that the bio-optical properties of water bodies, largely determined by CDOM and turbidity in large-river ecosystems, could be appropriate tools to describe the riverscape structure because of their optical contribution to spatial heterogeneity. First, these properties depend on the connectivity between the terrestrial and the aquatic parts of the landscape, then on the hydrological connectivity and mixing regime among the different portions of the watershed, which makes underwater light climate an integrative variable of upstream processes. Second, light composition and availability is a fundamental driver of the abundance and composition of photosynthetic communities at the base of aquatic food webs.

Picophytoplankton (0.2–2.0 µm) are major components of the photosynthetic biomass in many aquatic ecosystems, containing, for example, up to 70% of the carbon fixed annually in ultraoligotrophic waters [30], [31]. However, information about the spatial distribution of the prokaryotic and eukaryotic picoplankton community along environmental gradients, and the extent to which environmental factors regulate their dynamics, is still limited for inland ecosystems [32], [33]. Furthermore, information is especially lacking for river ecosystems, with studies presently limited to specific areas of the riverscape such as hydroelectric reservoirs [34] and river plumes [35]. The ecological significance of photosynthetic picoplankton has received increasing interest in recent years, and observations have led to novel insights concerning niche partitioning of ecotypes along gradients of proximal factors such as nutrients and light [8], [33], [36], [37]. Classically, nutrients (N and P) have been identified as selective factors for abundance of picoplankton [38], [39], but the relative abundances of red and green picocyanobacteria in lakes and oceans have also been shown to be related to underwater light color [8], [40]. Research has revealed a close correspondence between the absorption spectra of phototrophic microorganisms and the prevailing underwater light spectrum (refs in [8], [40]). Interaction of light and nutrients is thus likely to play a decisive role in structuring picophytoplankton communities in a mosaic of heterogeneous habitats with distinct niche availability. The spatial and temporal bio-optical heterogeneity that prevails in a large-river ecosystem thus offers a good opportunity to test the classical niche theory in a gradient of environmental conditions.

In that context, the objectives of the present paper were 1) to characterize, in a multiscale approach, the longitudinal and lateral hydrological connectivity patterns between river habitats and inflowing tributaries, 2) to identify the impact of tributaries on the distribution of water masses, 3) to model the spectral composition of the underwater habitats as a structural property of the riverscape and finally 4) to model the ecological consequences of spectral composition on the phytoplankton community structure. Here we show how the underwater light climate integrates well the physical structure of a complex and heterogeneous large-river system at the interface between land and ocean. We further show that the underwater light availability and composition that results from the connectivity among the different components of the SLR riverscape together constitute the main drivers of the abundance of major phytoplankton groups and their community composition.

## Material and Methods

### Study site and riverscape discontinuities

**Figure 1:**
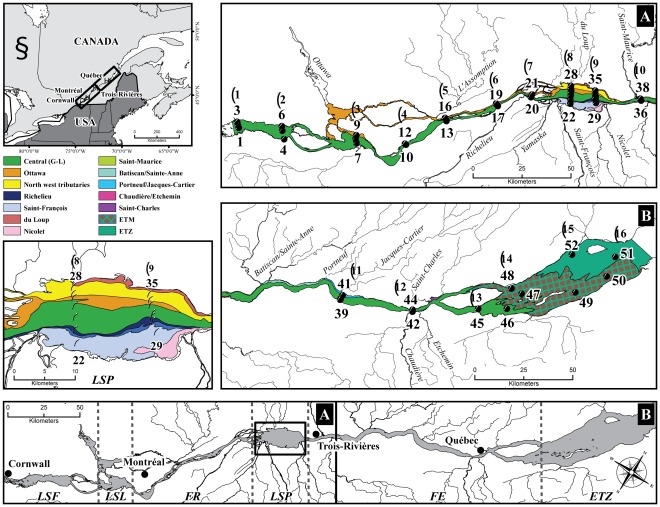
Distribution of water masses and sampling sites (1–51) for the St.Lawrence River. The study area is located from Cornwall to Île-aux-Coudres. Transect numbers (1–16) are indicated in circles. (A) Fluvial section including Lake Saint-François (LSF), Lake Saint-Louis (LSL), the fluvial reach (FR) and Lake Saint-Pierre (LSP). (B) Fluvial estuary (FE) and the estuarine transition zone (ETZ).

To keep emphasis on spatial patterns, we studied on the short time span of a week the SLR riverscape from its source at the outlet of the Great-Lakes, until the interface with marine waters at the estuarine transition zone (ETZ), 50 km downstream from the marine intrusion. No significant climatic events (e.g. high wind or rainstorms) occurred during the study. To better characterize the riverscape at fine and large scales, we reclassified the 450 km study area into six physical discontinuity zones (PDZs) (Fig. 1) based on landscape, bathymetric and hydraulic characteristics (Table 1). These are the three fluvial lakes, Lake Saint-François (LSF), Lake Saint-Louis (LSL), and Lake Saint-Pierre (LSP), the fluvial reach (FR) between LSL and LSP, the fluvial estuary (FE) flowing from LSP to the ETZ, and the ETZ,which represents the 50 km marine intrusion between the eastern portion of Île d’Orléans and Île-aux-Coudres.

**Table 1:** Riverscape characteristics of the physical discontinuity zones (PDZs): Lake Saint-François (LSF), Lake Saint-Louis (LSL), fluvial reach (FR), Lake Saint-Pierre (LSP), fluvial estuary (FE), and the estuarine transition zone (ETZ).

PDZ	Length	Width	Area of water (landmark)	Number of water masses	Number of tributaries	Volume	Z_m_	Watershedarea	Cumulated watershed area	Confluence density	Wetlandarea	Wetland	Proportion of photic zone
	Km	km	km^2^			km^3^	m	km^2^	km^2^		km^2^	%	%
LSF	50 (73**)	4.7 (1.3***)	225.4 (259.56)	2	3	1.12	4.97	772000	772000	0.06	20.43	9.06	100
LSL	26	6.5	136.31 (151.51)	3	3	0.62	4.55	148843	920843	0.13	6.85	5.03	60.32
FR	104	4.3	131.6 (253.24)	4	2	0.71	5.4	319940	1240783	0.025	12.01	9.12	57.70
LSP	35	15	241.02 (315.58)	8	5	0.75	3.11	21127	1261910	0.143	160.98	66.79	63.06
FE	182	3.2	472.38 (640.32)	10	9	4.23	8.95	68245	1330155	0.049	29.99	6.35	28.38
ETZ	58	22	1341.33 (1521.37)	2	1	14.96	11.15	2328	1332483	0.001	32.79*	2.44	16.95

* From Île d’Orléans to Tadoussac.

** Length including the Beauharnois Channel.

*** Channel length.

### Sampling design

**Figure 2:**
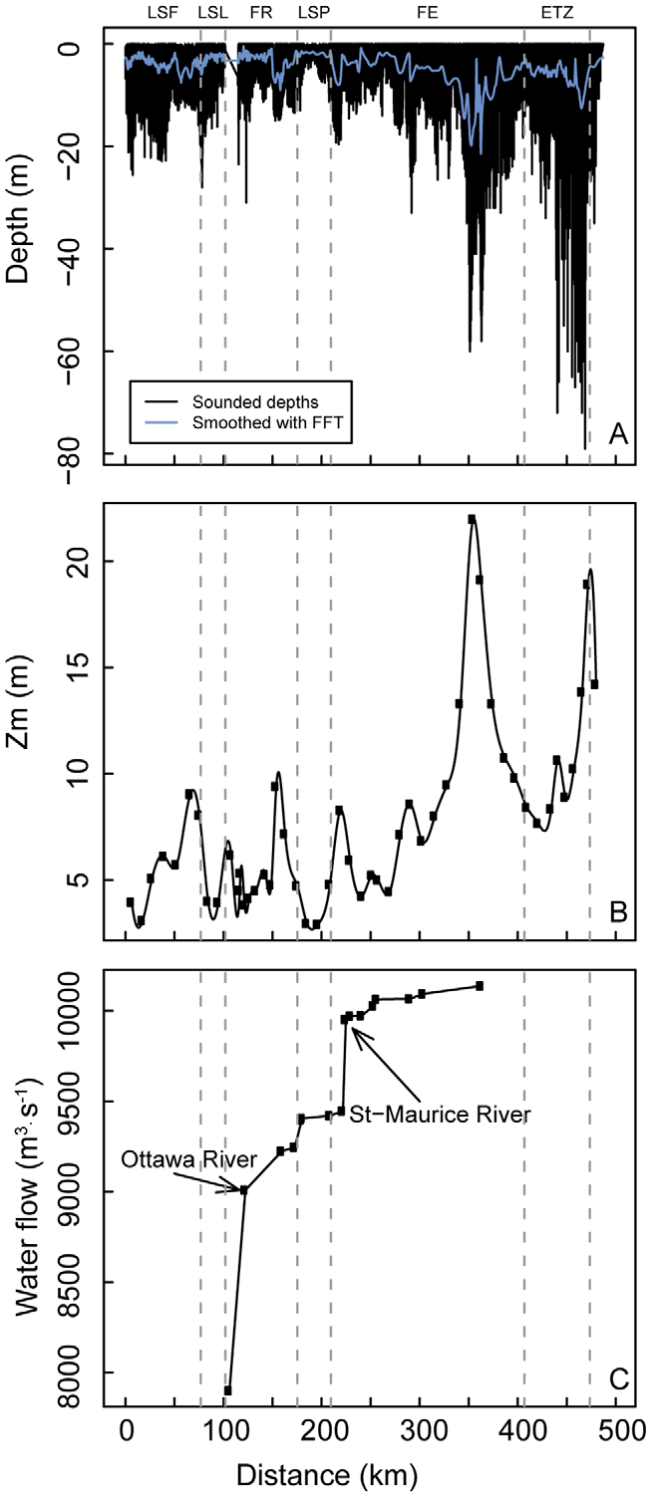
Distribution of water column depth, mean depth and water flow as a function of distance. (A) is sounded and smoothed depths, (B) is mean depth (volume/area), and (C) represents water flow from Cornwall to Île-aux-Coudres. Vertical lines are the boundaries of the physical discontinuity zones (PDZs): Lake Saint-François (LSF), Lake Saint-Louis (LSL), fluvial reach (FR), Lake Saint-Pierre (LSP), fluvial estuary (FE) and estuarine transition zone (ETZ).

Sampling was carried out aboard the RV *Lampsilis* from the Université du Québec à Trois-Rivières from August 8 to August 15, 2006. We collected a total of 51 samples, distributed along 16 transects along a 450 km downstream distance, from Cornwall to Ile-aux-Coudres (Fig. 2). Transects were positioned perpendicularly to the main east-west axis. We positioned the sampling stations in different water masses for each transect, based on Landsat-5 satellite imagery. In August the water levels were low and macrophytes were in their maximal growth phase [30]. Due to the low draught of the *Lampsilis*, we could access shallow depth zones (up to 1 m depth), which characterize the shallow fluvial lakes and coastal areas, including wetlands.

### Water mass distribution

The water mass distribution map (Fig. 1) used to determine the relative position of the vessel to each water mass was based on the analysis of Landsat-5 Enhanced Thematic Mapper (ETM) satellite images of the LSP taken between August 16^th^ and 26^th^ of 2006, using the approach described in [22]. Briefly, inland waters were differentiated according to their spectral properties using remote sensing with bands TM2 and TM3. Based on a similarity matrix of the per-pixel red-to-green surface reflection ratio, a clustering analysis, processed by an unsupervised clustering algorithm (ISODATA/PCI-Geomatica 10), was used to identify the specific lateral limits of each water mass. Stations that were positioned close to the junction of two water masses were validated according to their bio-optical properties to ensure their exact localization. Inspection of the SLR and major tributaries showed little variation in flows and water levels between the satellite image acquisition date and the dates of the sampling campaign, supporting the assumption of the relative stability of water masses during this period.

### Riverscape characteristics

#### Volume, surface, mean depth

We calculated the volume of the study zones of the SLR from bathymetric profiles measured by Fisheries and Oceans Canada (S-57 (ENC Electronic Navigational Charts) Hydrographic Data). The data showed a high degree of resolution, with a total of 21086 sounding measurements (minimum average of 0.2332±0.1854 km between the nearest neighbours). We then exported those points to ArcGiS *shapefile* format. Because bathymetric maps do not show sounding measurements in the dragged area (maritime channel), we exported the main channel delimitation as a polygon, which we then converted to points (*n* = 9021), to which Fisheries and Oceans Canada has assigned depth values of 11.3 m. To account for the temporal variation in hydrographic regime, we corrected the depth values for each zone using the water level measured during the corresponding sampling day. We determined the area under the influence of the correction factors using half the distance from the stations upstream and downstream of the current measurement station. We also calculated the area based on the landmark distribution. Along the longitudinal west-east axis, we used 19 measurement stations to perform the temporal correction in water level. Finally, we used a water mask to only keep the water volumes and surfaces associated with specific water masses. We then computed surfaces and volumes in ArcGiS 9.3 [41] using the 3D Analyst tool for each section of the SLR. We calculated mean depth as follows:
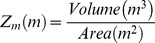
(1)The water mass characteristics, describing their length, area, flow rate, volume, mean depth (Z_m_) were calculated using the same procedure and appear in Table S1. Supplementary information about bathymetry and morphological characteristics is given in Text S2.

#### River network

The riverscape characteristics, including the morphological, bathymetric, and hydraulic records for each PDZ are presented in Table 1. We calculated a structural index of the river network in the SLR, the confluence density of inflowing tributaries, as in [19], for each PDZ. Thus:


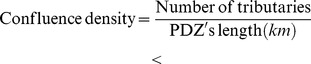
(2)

#### Spectral radiation and beam attenuation

Downward irradiance was measured at every 0.02 m with a spectroradiometer (HyperPro, Satlantic Instruments) which was slowly lowered through the water column to measure depth profiles of the cosine-corrected downwelling underwater irradiance (*E_d_*) at every 3 nm between 351 and 750 nm (100 wavebands) as in [42]. We equipped the Hyperpro with a C-star transmissiometer (Wet Labs Inc., 25 cm path length, λ = 660 nm) to measure depth profiles of the scattering of underwater particles (Tr) such as sediments. Light data were corrected automatically for “dark irradiance” values obtained from the shutter darks. These are continuously recorded during the measurements by occulting the input fiber with an optical shutter, typically after every five light samples. Further details are available in the Prosoft software user manual (Satlantic, Document SAT-DN-00228-Rev. C). Total irradiance (E*_d_*) corresponding to photosynthetically active radiation (PAR) (400–700 nm), blue (435–500 nm), green (520–565 nm), and red (625–740 nm) was calculated at each depth using equation [43].


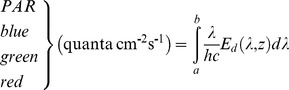
(3)

Where *h* is Planck’s constant, used to describe the size of quanta (6.62 × 10^-34^ J s), *c* the constant speed of light (299 792 458 m s^-1^) and *E_d_* (*λ*,*z*) the measured irradiance at wavelength *λ* at depth *z* (µW cm^-2^ nm^-1^). The parameters *a* and *b* represent the spectral bandwidths range used to calculate light intensity. The diffuse attenuation coefficients for blue, green and red spectra light, *K_d_* (m^–1^), were calculated by linear regression of the natural logarithm of light irradiances versus depth.

#### Conductivity, temperature, and turbidity

At each station, we used a multiprobe depth profiler (YSI, model 6600EDS-M, Yellowspring Inc.) to measure the conductivity, temperature, and turbidity of the water column. Values for the surface of the water columnwere averaged between 0.5 and 1.5 m.

#### aCDOM, DOC, and Chl a measurements

Water samples were collected at the surface (0.5 to 1.3 m) for all stations using a Go-Flow bottle (8 L) and immediately processed in the wet lab after collection. We filtered samples for the absorption coefficient of chromophoric dissolved organic matter (*a*CDOM) and DOC through Milli-Q-rinsed 0.22 µm Isopore membrane (Millipore) and stored them in the dark at 4°C until analysis. We measured CDOM absorption spectra in a 10 mm quartz cell at 1 nm intervals between 190 and 900 nm using a spectrophotometer (Shimadzu UV-2401PC) referenced against Milli-Q water. We used absorbance at 690 nm (where the temperature dependency is near zero) to correct the UV-absorption values. We converted absorbance values at 340 nm to absorption coefficients (*a*CDOM_340nm_) using the following equation [9]:


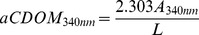
(4)

DOC was analyzed using high-temperature catalytic oxidation on an OI Analytical 1010 TOC analyzer using wet-persulfate oxidation.

#### Chlorophyll a

We filtrated duplicate subsamples of water on 25 mm–diameter, 0.7 µm poresize GFF filters (Millipore) immediately after collection and kept them frozen until analysis. Measurements were performed before and after acidification on a fluorometer (turner, model) after 24 h extraction with cold acetone [44].

#### Phosphorus analyses

At each site, we subsampled water in acid-washed borosilicate bottles for total phosphorus (TP) measurements. We filtrated samples for soluble reactive phosphorus (SRP) on 45 mm diameter, 0.7 µm poresize GFF filters (Millipore). We obtained concentrations of TP using the spectrophotometric determination of phosphates after digestion by persulfate, and we analyzed SRP using the acid molybdate technique [45].

#### <20 µm autotrophic phytoplankton and flow cytometry

We transferred water samples in 5 mL cryovials and fixed them with gluteraldehyde (0.1% final concentration) [46]. Samples were kept in the dark at 4°C for 10 minutes before being transferred to a cryofreezer (on board) at –80°C. Prior to analysis, we pre-screened samples on a 40 *µ*m nylon cell strainer. Samples were analysed using an Epics Altra flow cytometer (Beckman Coulter) equipped with a 488 nm laser (15 mW output) [47]. Forward-angle light scatter, right-angle light scatter, orange fluorescence from phycoerythrin (575±20 nm) (PE), and red fluorescence from chlorophyll (675±10 nm) were measured. 1.9 µm–diameter microspheres (Fluoresbrite plain YG, Polysciences) were added to each sample as an internal standard. Pico- (<2 µm) and nanophytoplankton (2–20 µm) were discriminated based on forward-scatter calibration with polystyrene microspheres of known size. With the flow-cytometer configuration used in the present study, we assessed phycoerythrin-containing cyanobacteria as picocyanobacteria, whereas we counted phycocyanin-containing cyanobacteria, if present, with the eukaryotic picophytoplankton as [47]. In order to test the accuracy of the flow-cytometric algal-count method, we compared pseudo replicates for the same cryovials analysed twice for 5 stations. The average coefficients of variation (root mean squared difference) varied between 1 and 8%. Our results showed this technique to be accurate.

#### Numerical analysis

We performed numerical analyses using the [R] graphical and statistical computing environment [48]. Before analysis, we normalized the data with a Box-Cox transformation [49] for statistical tests that required the assumption of normality. We verified colinearity among selected predictors using a variance inflation factor (VIF) with a threshold of 5.

We used asymmetric eigenvector map (AEM) modeling to take spatial connectivity into account and remove effect of spatial heterogeneity in order to better understand effects of environmental factors on pico-nano assemblage in the river.

The dataset used for numerical analysis was composed of three subsets. The dependent variables matrix (*Y*) consisted of PE cyanobacteria (pico- and nanocyanobacteria) collectively called ‘cyano’, eukaryotes (pico- and nanoeukaryotes) collectively called ‘euk’, cyano/euk (the ratio of pico- and nanocyanobacteria to pico- and nanoeukaryotes), and Chl*a*. Explanatory spatial variables (*X*_1_) consisted of six eigenfunctions (spatial variables) generated by the AEM analysis. The second set of explanatory variables (*X*_2_) contained environmental measurements (K*_d_*_ (PAR)_, SRP, TP, blue/red and green/red ratios, conductivity, temperature, transmittance and turbidity). We used loess curves to describe the pattern of changes with distance for the physical, chemical, and biological variables. This allowed us to identify the main tendencies in the longitudinal connectivity for each of the three main water masses.

#### Asymmetric eigenvector maps (AEM) analysis of connectivity

We generated the spatial variables used to model the lateral and longitudinal distributions of DOM components by asymmetric eigenvector maps (AEM) analysis [50]. Briefly, this technic is used to take into account the spatial connectivity in asymmetric systems such as we find in rivers. Subsequently, the spatial variables are used as covariables in statistical analysis to remove the effect of connectivity and hence remove the introduced spatial bias. The AEM directional graph of the river network was determined by associations between sampling sites according to the spatial distribution of the water masses (Fig. 1). Further details about the AEM modeling implementation can be found in [13].

To select for the significant environmental variables to be added to the spatial variables resulting from the AEM, we used the *leaps* [R] package to perform an exhaustive search for the best subsets of the environmental variables (K_d (PAR)_, SRP, TP, blue/red and green/red ratios, conductivity, temperature, transmittance and turbidity) for predicting the cyano/euk ratio in linear regression (Table 2), using an efficient branch-and-bound algorithm (i.e. an organized and highly structured search of all possible solutions). We selected the cyano/euk ratio specifically from among the other dependent variables because of its high degree of predictability and the integrative aspect of all biological size classes. However, since the algorithm returns a best model of each size, the results do not depend on a penalty model for model size (i.e. taking into consideration the number of selected variables) [51]. Therefore, to select the best model for predicting the cyano/euk ratio (Table 3), we used the Akaike Information Criterion (AIC) to compare and rank the models [52]. We assessed variation partitioning among spatial and environmental variables (i.e. percent of contribution of each predictor in the model) by hierarchical partitioning using the *lmg* method in the *relaimpo* [R] package, as described in [53].

**Table 2:** Prediction of color ratios based on the given scenarios.

Predictor	Model	Variables	AIC	Δ_i_	R^2^
* ***Green/red***	***1***	**Trans (33.1%) + ** ***a*** **CDOM** * **(50.7%)**	***−39.93***	***0***	***0.84***
	2	Trans	8.14	48.07	0.50
	3	*a*CDOM*	*−*10.78	29.15	0.68
					
* ***Blue/red***	4	**Trans (13.8%) + ** ***a*** **CDOM** * **(79.5%)**	***−54.86***	***0***	***0.93***
	5	Trans	51.50	106.36	0.26
	6	*a*CDOM*	*−*47.65	7.21	0.92

* Data have been log transformed.

**Table 3:** Best models (Δ_i_ AIC≤2) in the prediction of the cyanobacteria/eukaryote (cyano/euk) ratio using color ratios, K_d(PAR)_, and nutrient variables.

Model	Blue/red (%)	Green/red (%)	SRP (%)	TP (%)	K_d(PAR)_ (%)	R^2^	AIC	Δi
I	0.40		0.38			0.78	111.35	0
II	0.26	0.22	0.30			0.78	112.84	1.49
III	0.33		0.30	0.14		0.77	113.25	1.91
***IV***	***0.31***		***0.31***		***0.15***	***0.78***	***113.35***	***2***

## Results

### Structural riverscape elements

#### Physical discontinuity zones (PDZs)

Morphological, bathymetric and hydraulic characteristics of the PDZs appear in Table 1. The freshwater part (*sensu stricto* a river) accounts for the first 400 km and includes three fluvial lakes and two fluvial sections. The last 50 km consist of the marine-freshwater interface and include the maximum turbidity zone; it constitutes the sixth zone, the estuarine transition zone (ETZ), with salinities varying between 1 and 18 [54].

Riverscape characteristics (Fig. 2 and Table 1) reveal a discontinuous bathymetric (average depth and mean depth (Z_m_)) and morphological (length, width) profile of the different PDZs, primarily exemplified by large differences between the fluvial lakes and connecting sections (FR and FE). On the basis of landmark distribution, the three fluvial lakes (LSF, LSL and LSP) are typically large (4.7 to 15 km in width and 146.5 to 300 km^2^ in area) and generally shallow (mean depth from 3 to 5 m) (Table 1). The fluvial lakes dominated the total area of the river, with LSP (including the Sorel Islands) representing the largest of these lakes (300 and 241 km^2^ on the basis of landmark and water distribution, respectively (Table 1)). Wetlands generally occupy a high proportion (% of wetland/area for each PDZ) of these lakes compared with the fluvial sections FR and FE (Table 1). Fluvial sections cover a much longer upstream-downstream river distance (by a factor of 3 to 8) than do fluvial lakes (Figs. 1, 2). After LSP, the bathymetric profile increases in the FE until the ETZ. The FE is also the longest physical discontinuity zone, with a length of 150 km, which represents a third of the total studied distance. The rate of water flow typically increased in a gradient from upstream to downstream from LSL to the ETZ, with drastic increases in the discharge rate imposed by the inflows of the Ottawa and St. Maurice rivers (Fig. 2), which constitute the largest inputs of water, in terms of flow rates, from tributaries in the SLR.

The ETZ contrasts with the other PDZs in that it includes the presence of marine waters, varying between 1 and 18 in salinity, as well as a semi-diurnal tidal regime that, combined with the Coriolis force, imposes a complex hydrodynamic regime on the zone (refs. in [54], [55]). The ETZ has the largest area, volume, and mean depth of all PDZs, but the smallest percent of wetland area.

#### Tributaries as a structuring element of PDZs

A total of 23 tributaries with documented hydraulic records (BQMA Québec government; Hydro-Québec; Environment Canada), flow into the SLR between LSF and the ETZ (included) (Fig. 1). Small irrigation channels linked to agricultural activities are not documented. The highest confluence densities (number of confluents/PDZ channel length) of the PDZs are found in the fluvial lakes, with a maximum value of 0.143 in LSP, followed by LSL (0.130) and LSF (0.06) (Table 1). The confluence density and the impact of tributaries on water mass formation, called THI (tributary discharge rate/PDZ mean depth), were the major riverscape structural factors influencing the water mass distribution between the discontinuity zones and thus, ultimately, the physical heterogeneity of the SLR. A comprehensive description of the hydraulic characteristics of the tributaries and impact on water mass distribution throughout the PDZs is given in Texts S3 and S4.

#### Spatial changes in water masses distribution and environmental properties

**Figure 3:**
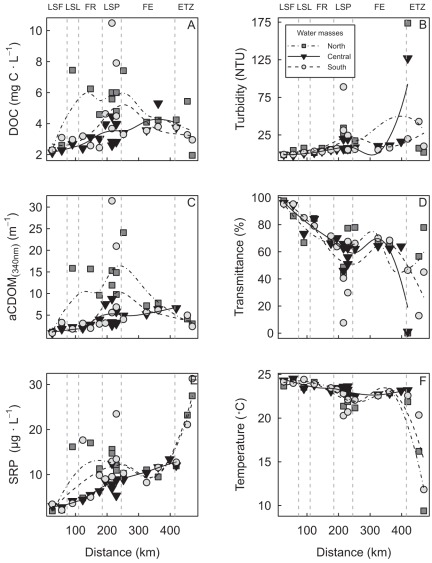
Distribution of environmental variables as a function of distance. (A) is DOC, (B) is turbidity, (C) is *a*CDOM, (D) is transmittance, (E) is SRP, and (F) is temperature from Cornwall to Île-aux-Coudres for the north, central, and south water masses. Vertical lines are the boundaries of the physical discontinuity zones (PDZs): Lake Saint-François (LSF), Lake Saint-Louis (LSL), fluvial reach (FR), Lake Saint-Pierre (LSP), fluvial estuary (FE), and the estuarine transition zone (ETZ).

To illustrate the connectivity patterns, we divided the river into three main water masses based on their lateral position relative to the Great-Lakes water mass: i.e., the north, the south, and the central GL water masses. Fig. 3 shows the high lateral heterogeneity among the water masses and the strong longitudinal continuity for the different physical and chemical variables in the center water mass; the north and south water masses were marked by discontinuities associated to tributaries inputs. More specifically, CDOM and DOC from the north and south water masses showed a sharp increase in concentration from LSF to LSP, followed by a significant decline thereafter, in the FE through the ETZ. Conversely, the central water mass exhibited a gradual and continuous increase in CDOM and DOC with distance, from LSL through the ETZ. Similarly, turbidity and transmittance values, which are inversely correlated, revealed a general increase in tripton (non-algal material such as suspended sediments) between LSF and LSP for all water masses, but this is followed by irregular changes afterward in the FE, and a drastic increase with maximal values in the maximum turbidity zone. SRP values and correlated TP (r^2^ = 0.31; not shown) from the north and south water masses showed a significant increase in concentrations from LSF to LSP, followed by a drastic increase from the mid-FE, with maximal values in the ETZ (Fig. 3). Conversely, the central water mass exhibited a gradual and continuous increase in nutrients with distance from LSL through the ETZ. For every variable, values were the lowest in the central water mass compared to the shore-most water masses impacted by terrestrial loading of matter via the proximate tributaries. We noted the highest nutrient values at stations located near point-source confluences (Figs. 1, 3), such as the Ottawa (north zone), Saint-François, and Yamaska (south zone) rivers or the water treatment stations of Montréal and Longueuil.

### Functional riverscape properties

#### Underwater spectral environment

**Figure 4:**
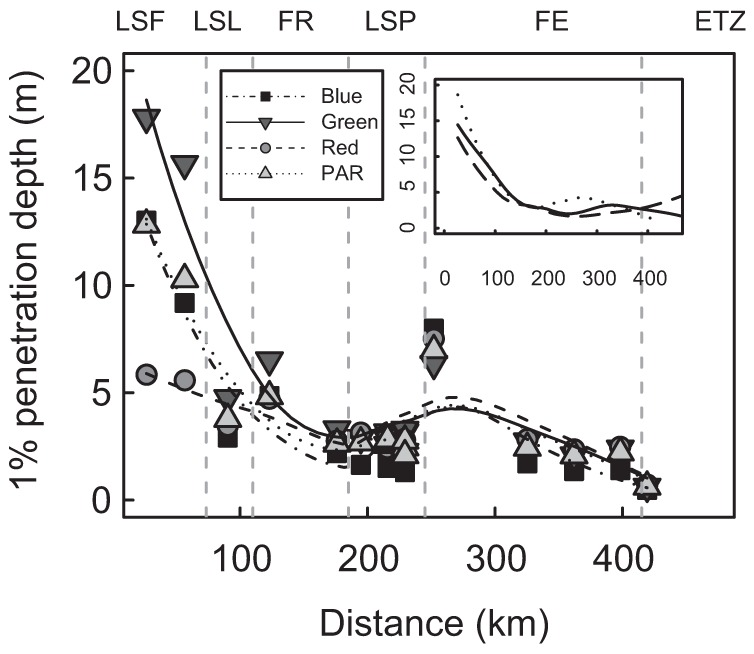
1% penetration depth of blue, green, red, and PAR wavebands for the central water mass. The study area islocated from Cornwall to Île-aux-Coudres. At the upper right, 1% penetration depth of PAR for the north, central, and south water masses as a function of distance.

These variations in CDOM and tripton abundance of water masses further determined their inherent optical properties which translated into variations in PAR attenuation and the underwater light spectra. PAR was generally present in 60% to 100% of the water column (calculated as (1% penetration depth (4.6/K*_d_*) divided by water column depth) * 100)) in fluvial lakes, as a result of their shallow depths (Table 1, Fig. 4), which generally favoured the growth of large macrophyte beds [26], [28]. Conversely, PAR was less available for autotrophs in the deeper FR, FE, and ETZ, where it occupied a smaller proportion of the water column (16% to 58%).

The 1% penetration depth of blue (435–500 nm), green (520–565 nm), red (625–740 nm) and PAR (400–700 nm) wavebands in all water masses (Fig. 4) showed an exponential decrease with distance from LSF to LSP (Fig. 4), consistent with increasing turbidity and CDOM(Fig. 3). We found the highest underwater light attenuations in the ETZ, and more specifically, in the maximum turbidity zone. On average, we found higher PAR attenuation in the north zone, followed by the south and the central (G-L) (Fig.4).

The attenuation of the different wavelengths was not homogenous over the lateral and longitudinal transects (Fig. 4). As a consequence, when expressed on the basis of relative color changes in the underwater column, i.e. the blue/red and green/red ratios, these displayed a general exponential decrease from LSF through the outlet of LSP, followed by a subsequent flattening of the curve in the FE, expressing minimal changes with distance after LSP (Fig. 4). This trend contrasted significantly with the underwater color expressed on a distinct basis instead of a relative basis, especially in LSP and transects 10 (Fig. 4). Color ratios subsequently increased in the ETZ. Moreover, the underwater color spectra exhibited specific signatures within each water mass, with blue/red showing maximum values in the central water mass (G-L), followed by the south and the north water masses, respectively. Conversely, the green/red ratio was maximal in the south, followed by the central and the north.

**Figure 5:**
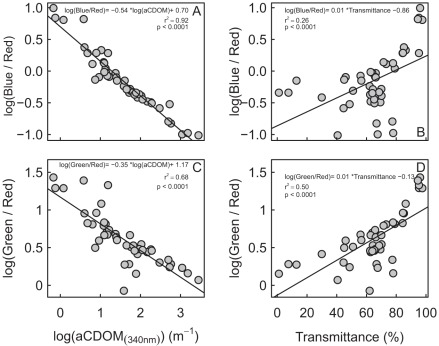
Relationships between the color ratios and inherent optical properties of the water column. Relationships between blue/red ratio and (A) *a*CDOM and (B) transmittance. Relationships between the green/red ratio and (C) *a*CDOM and (D) transmittance.

**Figure 6:**
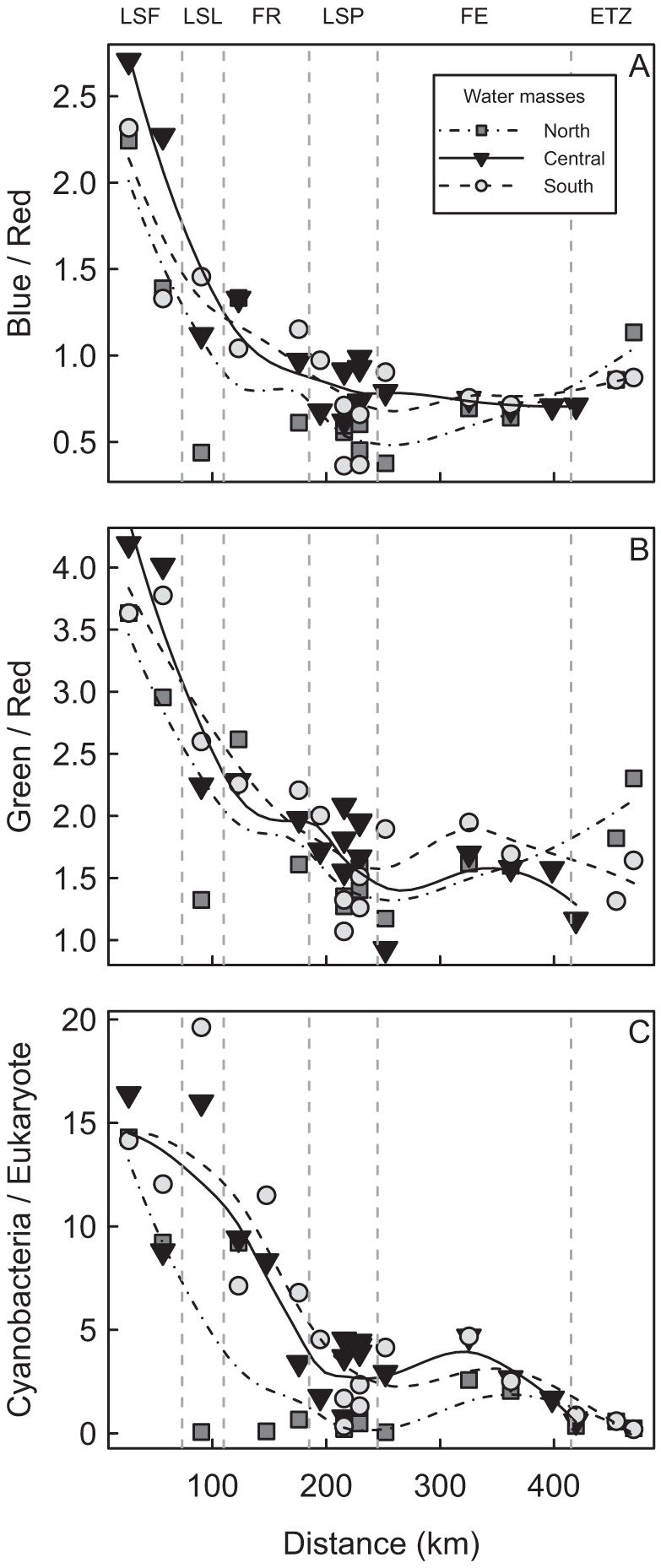
Distribution of color ratios and phytoplankton as a function of distance. (A) is blue/red, (B) is green/red, and (C) is cyanobacteria/eukaryote ratios from Cornwall to Île-aux-Coudres for the north, central, and south water masses. Vertical lines are the boundaries of the physical discontinuity zones (PDZs): Lake Saint-François (LSF), Lake Saint-Louis (LSL), fluvial reach (FR), Lake Saint-Pierre (LSP), fluvial estuary (FE), and the estuarine transition zone (ETZ).

The underwater light color closely matched the *a*CDOM and tripton (transmittance and turbidity) distribution within these three main water masses (Fig. 3), indicating the presence of a spectrally dependent attenuation gradient. This gradient is illustrated by the fact that at increasing concentrations of *a*CDOM and tripton, the blue/red and green/red ratios revealed a shift in underwater light color from the blue and green part toward the red part of the spectrum (Figs. 5 and 6). AIC analyses conducted on color ratios and environmental variables (transmittance and *a*CDOM) showed that the *a*CDOM and transmittance (tripton) values were the best variables for predicting the green/red and blue/red ratios, explaining 84% and 93% of variance, respectively (Table 2). *a*CDOM was the best predictor for both ratios, followed by transmittance (tripton). However, the percent contribution of *a*CDOM to color attenuation (Table 2) was higher for the blue/red (r^2^ = 0.795) than the green/red ratios (r^2^ = 0.507). Conversely, the tripton (transmittance) contribution to color attenuation was higher for the green/red ratio (r^2^ = 0.331) than for the blue/red ratio (r^2^ = 0.138). Tripton and *a*CDOM thus acted differently on the underwater light color spectrum, and both bring relevant information to the prediction of color ratios.

#### Pico and nanophytoplankton community

**Figure 7:**
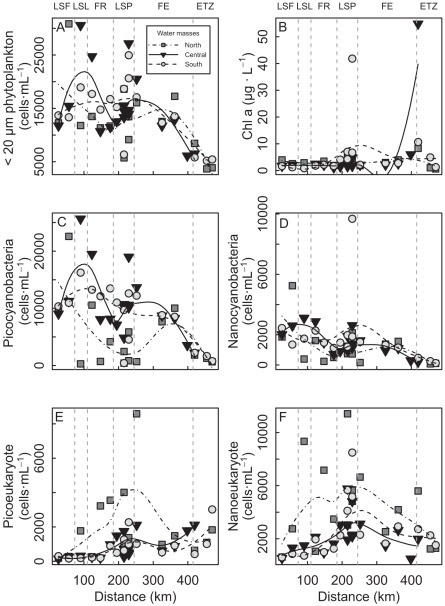
Distribution of phytoplankton and Chl *a* as a function of distance. (A) is <20 µm phytoplankton, (B) is Chl *a*, (C) is picocyanobacteria, (D) is nanocyanobacteria, (E) is picoeukaryotes, and (F) is nanoeukaryotes from Cornwall to Île-aux-Coudres for the north, central, and south water masses. Vertical lines are the boundaries of the physical discontinuity zones (PDZs): Lake Saint-François (LSF), Lake Saint-Louis (LSL), fluvial reach (FR), Lake Saint-Pierre (LSP), fluvial estuary (FE), and the estuarine transition zone (ETZ).

Total Chl*a* biomass showed high concentrations only in LSP and the ETZ. It remained relatively low (on average) between LSF and LSP (Fig. 7). Values increased thereafter in LSP, near the north and south tributary confluences, decreased in the FE, and reached maximum values (>50 µg/L) in the maximum turbidity section of the ETZ.

The picoplankton fraction generally dominated numerically the <20 µm phytoplankton, with a mean relative abundance of 65% (Fig. 7). Within the picoplankton sizeclass (0.2–2.0 µm), the picocyanobacteria were the most abundant cells, contributing an average of 81%, whereas picoeukaryotes represented only 19% of the corresponding number of cells. The relative contribution of picoplankton to the <20 µm phytoplankton generally decreased from LSF to the ETZ, indicating a gradual replacement of picoplankton by larger-sized nanoplanktonic eukaryotes (Fig. 7).

The total number of <20 µm cells generally decreased from LSL to the ETZ (Fig. 7), with maximal abundance near the confluence of tributaries such as the Yamaska, St-François and Richelieu rivers in LSP (Fig. 1, Stations 29, 31, 37) and near the water-treatment inflows of Montreal (Fig.1, Stations 8, 11) in LSL and the FR. Picocyanobacteria were generally less abundant in the CDOM- and nutrient-rich water mass from the north of the SLR and dominated in the central and south water masses, with maximum densities near the confluence of tributaries, as for the <20 µm cells. Conversely, picoeukaryotes dominated numerically in the north of the SLR, with maximum densities in the CDOM- and nutrient-rich water masses such as the Ottawa, L’Assomption, Saint-Maurice, and West tributaries. They were also abundant in the CDOM-rich Saint-François and Yamaska water masses in the southern portion of LSP.

#### Cyanobacteria, eukaryotes, underwater light regime, and nutrients

The ratio of cyanobacteria to eukaryotes (cyano/euk) showed an exponential decrease from LSF to the ETZ (Fig. 6), with maximum changes between LSF and LSP. Each group of cells showed an opposite pattern of distribution, with cyanobacteria and eukaryotes decreasing and increasing, respectively, with distance from LSF to the ETZ (Fig. 7). This pattern of distribution matched very closely the underwater light regime, with a high correlation between the cyano/euk ratio and the blue/red (r = 0.75) or green/red (r = 0.71) underwater wavelengths. We found a similar significant relationship between cyano/euk and nutrients (PO_4_ (r = –0.79) and TP (r = –0.59)).

#### Connectivity with the ETZ

During the transition from freshwater to saltwater, we observed drastic changes for all studied variables at all stations, but the strongest changes were in the north, due to the maximum marine intrusion in that area, linked to the Coriolis force (Fig. 2). The maximum turbidity zone (Fig. 1) typically shows the maximal turbidity values and a corresponding decrease in PAR (Fig. 4). SRP (and TP, not shown) values generally increased abruptly, and temperature dropped. CDOM and DOC generally decreased throughout the ETZ.

The total number of <20 µm cells generally decreased in the ETZ. Pico- and nanocyanobacteria, the percent of PE-rich cells (not shown) and the cyanobacteria/eukaryote ratio generally dropped throughout the ETZ. Pico- and nanoeukaryotes showed no decreasing trend with marine intrusion.

#### Spatial and environmental models

**Figure 8:**
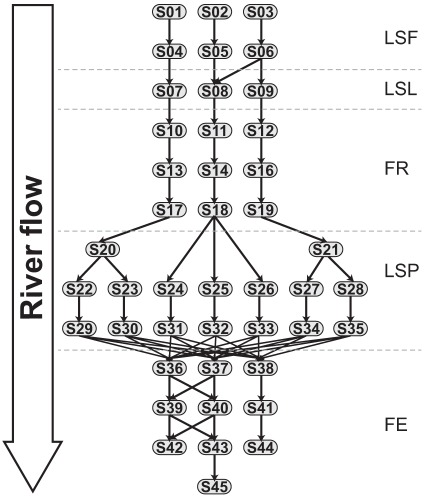
Directional graph representing the connectivity process of the SLR riverscape between Cornwall and Ile-aux-Coudres (ETZ). Numbers in circles represent sampling sites.

The connectivity diagram at the base of the AEM analysis appears in Fig. 8. Stations from the ETZ were excluded from the analysis because of the distinct tidal and hydrodynamic regime that prevails in that PDZ and also to eliminate the dilution effect associated to the intrusion of marine water, from the spatial analyses that were aimed at testing the direct effect of connectivity. AEM analysis produced a total of 41 eigenvectors, of which 15 have been retained by the variable selection analysis (see [13] for further statistical details). Those 15 AEM variables were kept to model the spatial distribution of the cyano/euk ratio and of the pico- and nanocyanobacteria and eukaryotes. Environmental variables selection analysis pointed out that blue/red, green/red, PAR and SRP were the best predictors for the cyano/euk ratio and of the pico- and nanocyanobacteria and eukaryotes (Table 3).

**Figure 9:**
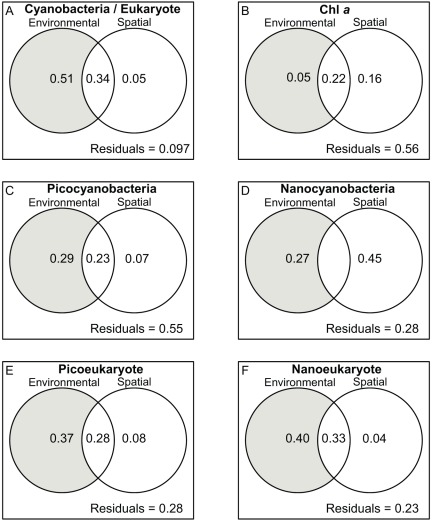
Variation partitioning between spatial and environmental models. (A) is the cyanobacteria/eukaryote ratio, (B) is Chl *a*, (C) is picocyanobacteria, (D) is nanocyanobacteria, (E) is picoeukaryotes, and (F) is nanoeukaryotes. The shaded area represents the environmental variation once the spatial influence has been removed.

The combination of spatial and environmental models predicted the different planktonic groups to a high degree, varying between 45% and 90% (Fig. 9). Variation partitioning among spatial and environmental variables showed that the spatial influence *per se* on the dynamics of the planktonic groups (advection only; right circles in Venn diagrams) generally accounted for a small fraction of the total explained variance, varying between 4% and 16% in five of the six models. However, for the nanocyanobacteria model (Fig. 9D), spatial contribution reached 45%. On the other hand, a large part of the total variance explained by the environmental model was found to be spatially structured, i.e. under the influence of upstream environmental conditions. Furthermore, this common part of explained variance between environmental and spatial variables (overlapping regions in Venn diagrams) varied between 0% and 34% (Fig. 9).

**Figure 10:**
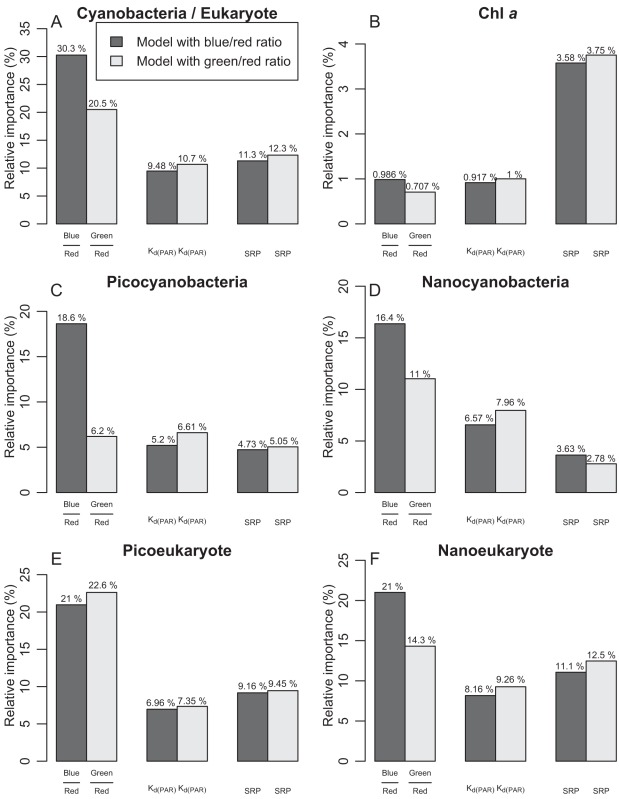
Relative importance of each independent variable for the models with color ratios. Data represents the despatialized environmental fraction for the models with the blue/red and green/red ratios. (A) cyanobacteria/eukaryote ratio, (B) Chl *a*, (C) picocyanobacteria, (D) nanocyanobacteria, (E) picoeukaryotes, and (F) nanoeukaryotes.

Local environmental conditions (the pure local conditions not influenced by upstream flow – shaded areas in Venn diagrams, Fig. 9) generally had more impact than spatial connectivity on the distribution of the different planktonic groups. However, we found Chl*a* and nanocyanobacteria to be preferentially driven by the asymmetrical gradient i.e. the connectivity found in SLR (Figs. 9B and 9D). In order to identify the specific roles of environmental variables on the biological variables, we calculated the relative contributions of each predictor, once spatial influence was removed (shaded areas in Venn diagrams), for two scenarios (Fig. 10). Both models included K_d(PAR)_ and SRP as common predictors and varied with blue/red ratio (black bars) and green/red ratio (gray bars). Except for the picoeukaryote model (Fig. 9E), we found the blue/red ratio to have the highest predictive power in all models. Additionally, in all models except model B, color ratios were the best of the three predictors, whereas nutrients (SRP) played a stronger role in the prediction of Chl*a*, illustrating that nutrients supported high biomasses while light quality affected community composition (Fig. 10). Picocyanobacteria showed the highest difference between blue/red and green/red ratios over all the models, followed by cyano/euk ratio, nanoeukaryotes, and nanocyanobacteria. In all cases, K_d(PAR)_made a higher contribution to the model when used in combination with the green/red ratio, but its contribution remained generally low varying between 0.9% and 10.7% of explained variance.

## Discussion

### Riverscape structural eements

#### Connectivity as a structural element of the riverscape

**Figure 11:**
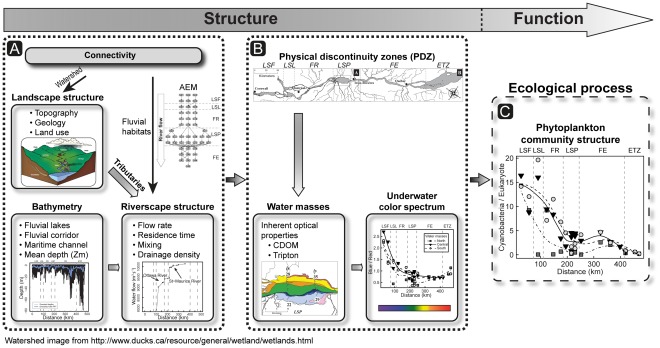
Conceptual model of the St. Lawrence riverscape’s structure and functioning. The degree ofconnectivity and thus transfer of material between habitats in the SLR is determined by the spatial structure of the tributary network and morphology of the riverscape (Fig. 11 panel A). The matter transported by the tributary network interacts with the receiving river’s morphological characteristics (e.g. shape and mean depth), which ultimately redistribute the transported matter in a given direction. The structure of the drainage network is the dominant factor in determining physical heterogeneity through the water mass distribution and the type and amount of injected material on a multikilometer scale (Fig. 11 panel B). This induces the formation of distinct water masses with contrasting underwater light characteristics (Fig. 11 panel B). Ultimately, this optical heterogeneity acted as an important driver in the distribution of the phytoplankton community structure (Fig. 11 panel C).

The SLR flows at the lowest point in the landscape and becomes a processor for a large variety of carbon and nutrient sources from the inflowing tributaries, which integrate the exchanges among lakes, reservoirs, lower-order rivers (streams) and their surrounding terrestrial environments. Subsidies injected from tributaries add to the pool of local carbon and nutrients advected from upstream or released from wetlands. Confluences and watershed characteristics have already been recognized as key elements of physical heterogeneity in rivers (refs. in [19], [20], [22], [23]). Actually, tributary-induced spatial and temporal heterogeneity in resources and habitats has been proposed as representative of biological hotspots within a river network because of its association with increased local richness [19], [56], despite the still-limited empirical evidence demonstrating the ecological importance of morphologically diverse tributary junctions. Our results complement this established pattern and expand the biological effects of confluence to a much larger scale than initially proposed, with the discovery of tributary-induced water masses distributed between 6 and 150 km downstream from the confluence site and characterized by distinct environmental properties. This study thus emphasizes the key role played by tributaries in the riverscape, through the formation and disappearance of water masses (Fig. 1) and their resulting impact, at multikilometer scales, on the river’s environmental conditions–such as the underwater color spectrum–and ultimately on the biology of phototrophic microorganisms (Fig.11).

#### Connectivity at different scales: a riverscape perspective of physical processes

Riverscape ecologists have emphasized the importance of studying riverine habitats and their patchiness over scales of many kilometers and, thus, also on a global scale [19], [24]. This study is, to our knowledge, one of the first attempts to characterize large rivers at a multiscale riverscape perpective.

We observed strong horizontal gradients in the SLR for physical and chemical environmental properties on both the longitudinal and lateral axes, and this gradient operates between habitats at both broad and fine spatial scales (Fig. 3). The first part of the following discussion refers to the freshwater part (i.e. the first 400 km) of the river (*sensu strictu* a river), since the intrusion of marine waters farther downstream presents a different hydrodynamic and tidal regime [57]. The ETZ connectivity will be discussed separately farther below.

On a *large* spatial scale (>20–450 km), our results demonstrate that for the central water mass in the SLR, physical and chemical variables generally exhibited continuous, although nonlinear, downstream changes (Figs. 3, 4), thereby representing a continuum in environmental variables (*sensu* [58]). This continuous nonlinear pattern could be explained by the progressive lateral transfer of matter through mixing with the accompanying (tributary-induced) nutrient, DOC and CDOM water masses and riparian zones, in combination with the strong hydrologic forcing imposed by the increasing discharge rate in the downstream direction.

On a *small* spatial scale (1–5 km), the lateral water masses (north and south) behaved much differently than the central water mass, showing discontinuities at fine (1–5 km) and medium (>20 km) scales, both transversely and longitudinally, in the environmental variables and related inherent optical properties. The environmental variables’ slope of change with distance generally increased from LSL to LSP, but decreased drastically thereafter in the FE, in response to the lower inputs of matter from tributaries that resulted from the reduced density of confluences and drainage. Among these variables, SRP (and correlated TP) and CDOM values proved to be good tracers of point-source connectivity, showing their highest values at the tributary junctions and locations of point-source pollution, such as the urban sewage treatment outlets at Montreal and Longueuil (Fig.1).

Our results reveal that the SLR is largely heterogeneous and characterized by a series of physical discontinuities operating at small and large scales and largely controlled by the interactions of morphology and the tributary network, over which the hydrodynamic regime is superimposed. These discontinuities constitute hydrogeomorphic “patches” that modify environmental conditions in agreement with the model in [16], called “riverine ecosystem synthesis” (RES). The RES model disagrees with the model in [58], called the “river continuum concept,” which proposed instead that rivers operate as gradients of continuous longitudinal change, where upstream processes continuously induce predictable processes downstream. However, these models were, when published, solely conceptual. Our study validates the model in [16] through the use of an exhaustive empirical data set and convincingly rejects the continuum [58] model, instead supporting the discontinuity view of rivers, as previously discussed by Poole [59] (and references therein).

#### Connectivity and underwater light spectrum

The underwater light spectrum proved to be the most integrative physical variable of spatial connectivity and representative of the changes in CDOM and tripton (non-algal material) over the course of the SLR. This translated into a rapidly changing and heterogeneous large-river system where the tributary-induced inputs of CDOM and tripton were responsible for a spectral shift from blue to green to red underwater wavebands and, consequently, for a shift of pico- and nanoprokaryotes to pico- and nanoeukaryotes. CDOM and tripton affected the color spectra differentially in terms of strength of attenuation and of the wavelengths affected. The percent contribution of CDOM to color attenuation was higher for the blue/red than for the green/red ratios, an observation that agrees with the increased attenuation of CDOM in the UV and blue parts of the spectrum [9], [20], [22]. Tripton showed the opposite pattern of color attenuation, with higher attenuation in the green/red than in the blue/red, which also agrees with increased scattering, and thus attenuation, at higher wavebands by non-living material [54]. These observations highlight the large differences between the bio-optical properties of large-river ecosystems in comparison with pelagic marine waters, caused by the impact of terrestrial-aquatic exchanges on the light-absorbing constituents. However, it shows similarity with coastal marine areas [60], [61] and especially the SLR estuarine system [62], where the CDOM absorption strongly surpassed particle absorption, thus contributing differentially to the water color.

#### Impact of the underwater light spectrum on phytoplankton abundance and community structure

On a *large* spatial scale, the phytoplankton community mimics the connectivity patterns observed for the environmental variables, with the <20 µm cells generally decreasing downstream in a continuous although non-linear fashion (Fig. 7), thereby representing a continuum of biological variables. However, a finer analysis of the community structure reveals that the prokaryotes and eukaryotes vary in opposite directions, responding antagonistically to the same environmental variables. Furthermore, the SLR’s ETZ behaves as a gate to the ocean, a description that emphasizes the key role played by landscape and riverscape connectivity (both lateral and longitudinal) through the advection and accumulation of freshwater-originating phytoplankton, nutrients and DOC into the ETZ, and through supporting the enormous secondary productivity both in that zone and downstream, even as far as 200 to 400 kilometers (see [54], [55]).

On a *small* spatial scale, the lateral water masses (north and south) behaved much differently than the central water mass, showing discontinuities in the phytoplankton distribution at fine (1–5 km) and medium (>20 km) scales, both transversely and longitudinally, in the composition of the community structure. For instance, the north water mass was strongly populated by the eukaryotes and exhibited the lowest numbers of prokaryotes. Conversely, the central and south water masses were densely inhabited by the prokaryotes. This exemplifies the need to consider riverscape studies from a *multiscale* perspective in order to truly understand both the regional and local structural connectivity and functional spatial variations that are representative of a multiscale approach to landscape ecology.

#### The role of connectivity and environment in controlling phytoplankton variation partitioning: removing the influence of spatial connectivity

The AEM analysis revealed that both spatial and environmental models predicted the planktonic community structure with a high degree of determination, showing the importance of connectivity processes in controlling the availability of proximal factors, which in turn determines the phytoplankton community structure. The unique influence of the asymmetric spatial gradient (i.e. direct transport of the cells from upstream or laterally) generally explained little of the variation in planktonic structure (right circles in Venn diagrams, Fig. 9). Those results suggest the contribution of suspended cells to local communities by passive drifting from upstream (including the Great Lakes) is small overall, and confirm the presence of structural environmental conditions influencing phytoplankton dynamics in the SLR.

The environmental variables, on the other hand, were spatially structured, as evidenced by the high contribution of the central part (overlapping area) of the Venn diagrams (Fig. 9), which shows the interaction of the spatial component and the environmental structure. The spatial structure of environmental variables has been observed before (e.g [17], [63]), and these studies have shown that connectivity is a strong driver of local environmental conditions, even when connectivity was not a strong driver of phytoplankton per se. To further identify the specific environmental drivers of phytoplankton, we tested the relative contribution of local environmental conditions, that is, once spatial influence was removed, to the planktonic groups and to Chl*a* distribution. The pure local environmental conditions, not influenced by upstream or lateral flow (left shaded circles in Venn diagrams, Fig. 9), generally had more impact on the different planktonic groups and explained most of their distribution. In all models, color ratios (green/red, blue/red) explained most of the variance. As much as two or three times as much variance was explained by underwater light climate as was explained by nutrients or temperature, which contributed the least to the distribution of the phytoplanktonic groups. Together, these results emphasize the key role of light among environmental and hydrological drivers in determining the size and composition of algal populations in large rivers.

### Proximal factors of phytoplankton community structure

#### Nutrients and temperature

Nutrients and temperature are the most commonly reported drivers of phytoplankton communities in a multitude of aquatic and marine systems (e.g. [64], [65]). These proximal factors of photosynthesis operate in concert with light at the level of organismal growth and are also involved in the functional assemblage of phytoplankton through adaptive constraints on species assemblages [65], [66]. Additionally, resource competition theory provides a useful framework for understanding phytoplankton diversity dynamics [67].

A recent study by Somogyi et al. [68] showed a competitive advantage for picoeukaryotes during winter’s low light and temperature conditions (<15°C), while higher temperatures and light intensity were more favorable for picocyanobacteria. Several studies have revealed that nutrients differentially affect the picoplankton community by decreasing prokaryotic cell numbers and increasing eukaryotic cell numbers. Schallenberg and Burns [69] have proposed picoplankton as an early indicator of ecosystem eutrophication. They observed a reduction in the growth rate of red picocyanobacteria with high phosphate concentrations during bioassays. In Lake Tahoe [33], picoeukaryotes were more abundant near the nitrocline, and thus near increased nutrient concentrations. Similarly, Wang et al. [34] noticed a reduction in (red) PE-picocyanobacteria with eutrophication in dam reservoirs, but picoeukaryotes showed no significant variation. Maximum abundance of picoeukaryotes has been observed during spring mixing and nutrient-replete conditions [32].

Our results, however, do not support such a contribution of temperature and nutrients to shaping phytoplankton communities. To test whether temperature had a significant effect on picoplankton and cyano/euk, we tested both models independently and used the AIC criterion to determine if it was necessary to include this variable in the models. Further, despite a general decrease in cyano/euk with increasing nutrient concentrations, our results indicate that for the SLR, it is in fact a spatial effect of downstream connectivity that controls nutrients and phytoplankton simultaneously.

#### Underwater light spectrum

Our results demonstrate that the composition of the underwater light environment, i.e. the relative proportions of the diverse colors of the light spectrum, was the strongest predictor of the phytoplankton community structure. This corroborates decades of research in marine bio-optics (e.g. [9], [70]), where remote-sensing data has been used to quantify phytoplankton biomass in terms of Chl*a* (e.g. [71]) or to identify target species such as taxa associated with harmful algal blooms (e.g. [72]–[74]) based on the match between reflectance colors and algal pigment composition (more refs in [75], [76]).

More specifically, in the spectrally heterogeneous SLR, the spectral shift from blue to green to red underwater wavebands was correlated with a shift from prokaryotes to eukaryotes for both pico- and nanoplankton fractions. These observations support earlier studies [77], whose authors found, under laboratory conditions, similar effects of light quality and intensity on the photosynthesis and growth of marine eukaryotic and prokaryotic phytoplankton clones growing under various spectral light fields. The prokaryotic, phycoerythrin-rich *Synechococcus* grew better under green light, while eukaryotes achieved higher abundance under blue. Similarly, Stomp et al. [8] observed a spectral shift from the blue part toward the green and red parts of the light spectrum with increasing gilvin (CDOM) and non-living material (tripton) concentrations in different freshwater and marine ecosystems. They found a higher abundance of eukaryotes in a turbid, tripton-rich peat light, which absorbed the available red light with pigments such as Chl*a* and *b*. However, in the Baltic Sea, where the CDOM and tripton were intermediate, the red-colored (PE) picocyanobacteria of the genus *Synechococcus* absorbed the available green light with its pigment phycoerythrin. Furthermore, a comparison of the growth response of two freshwater strains of *Synechococcus* sp., one PE cells and the other phycocyanin (PC) cells, demonstrated the selective value of red light in stimulating PC and suppressing PE [78]. Blue and green light were used more efficiently than red wavelengths of similar intensity by PE-*Synechococcus*. Similarly, Pick and Agbeti [79] compared oligotrophic lakes with colored ones and found that the contribution of eukaryotic picoplankton to the total picoplanktonic biomass increased with the higher PAR extinction coefficient in colored lakes. They also observed a decrease in the percentage of phycoerythrin-containing picoprokaryotes with increasing light attenuation, which they attributed to the changes in spectral quality that occur as lakes become more colored by humic material [80]. Craig [81] also noticed the prevalence of eukaryotic over prokaryotic picophytoplankton in less transparent eutrophic lakes. More recently, a higher abundance of picoeukaryotes was measured in turbid soda pans with low PAR intensities during winter [68].

We also observed a negative correlation between PAR availability and total number of planktonic autotrophs from LSF to the ETZ, in agreement with previous seston biomass (Chl*a)* diminution between upstream and downstream areas of the SLR [26], [28]. However, PAR alone was not as good as light color spectra for explaining the observed changes in the phytoplankton community structure. For instance, the variation partition analyses conducted on cyano/euk and environmental variables showed that K_d(PAR)_ contributed at most 15% for the only model where the K_d(PAR)_ contribution was retained, in comparison with color ratios, which explained 26% and 40% of the variation of community structure (Table 3). Among all underwater light spectra, the blue/red ratio is the predominant factor contributing to the color environment suitable for the cyano/euk community. Similarly for picoplankton, the blue-red ratio contributed 3- and 10-fold more to the total explained variation in densities of picocyanobacteria and picoeukaryotes, respectively, than did PAR.

Therefore, this study validates other laboratory and field evidence that has conclusively demonstrated the variation in relative abundances of size-fractionated prokaryotes and eukaryotes with the underwater light spectrum across many ecosystems, including clear oceans [8], [36], [40], coastal waters [82] and lakes [83]. However, to our knowledge, information about the distribution of picoplankton, and especially picoeukaryotes, along environmental gradients is generally lacking for river ecosystems, with available research limited to specific areas of the riverscape such as hydroelectric reservoirs [34] and river plumes [35], which are not representative of the whole riverscape. Moreover, available studies in running waters do not provide information about the mechanisms that control underwater light spectra and the impact of those light spectra on phytoplankton dynamics. Our results conclusively demonstrate the proximal impact of watershed properties on the underwater spectral composition in a highly dynamic river environment characterized by unique structuring properties such as a high directional connectivity, numerous sources and forms of carbon [13], and a rapidly varying hydrodynamic regime. Furthermore, in the SLR the low phytoplankton biomass did not contribute significantly to structuring the underwater color spectrum, in contrast with CDOM and tripton (non-algal particles). This emphasizes that light is a driver of phytoplankton function and not the opposite; that is, the phytoplankton do not cause the underwater light environment, a situation that is often observed in marine pelagic environments [9].

### From riverscape structure to riverscape function: a multiscale interaction between light properties and phytoplankton

In summary, this study demonstrates that tributaries constitute a key landscape element interacting with riverscape attributes and structuring the riverscape’s environmental conditions. Tributaries exert a profound impact on the receiving river through changes to the inherent optical properties of water masses (caused by, for example, CDOM, transmittance and turbidity), underwater color spectra and other proximal factors (e.g. nutrients) of photosynthesis, which in turn determine the phytoplankton community structure (Fig. 11).

### Conclusion

Our results emphasize the general need to characterize the underwater light availability in rivers and streams, as formerly addressed by Davies-Colley and Nagels [15] for running waters. We surmise that the underwater spectral composition represents a key integrating and structural property of large, heterogeneous river ecosystems and a promising tool to study autotrophic functional properties such as phytoplankton community structure (this study), primary production [75], trophic transfer efficiency [84] or ecosystem metabolism. It confirms the usefulness of using the riverscape approach to study large-river ecosystems and initiate comparison along latitudinal gradients. Riverscape-functioning studies at a multiscale level thus constitute a new and challenging field of research, which will benefit from interdisciplinary work at all trophic levels.

## Supporting Information

Figure S1 **Drainage area versus average discharge rate for the 165 largest rivers in the world. The SLR appears as a black circle.**
(EPS)

Table S1 **Hydraulic and landscape characteristics of the 23 main tributaries flowing into the SLR between Cornwall and Île-aux-Coudres.** Flow rates were measured on a daily basis during the sampling period (August 8–15, 2006); n/a refers to non-available data.
(DOCX)

Table S2 **Water mass characteristics, describing their length, area, flow rate, volume, mean depth (Z_m_), and tributary hydrological index (THI) expressed as the flow rate of tributary/Z_m_.**
(DOCX)

Text S1 **The St. Lawrence River as a representative model of large river ecosystems.**
(DOCX)

Text S2 **Bathymetry and morphological characteristics.**
(DOCX)

Text S3 **St. Lawrence River network characteristics.**
(DOCX)

Text S4 **Structure of the drainage network and physical heterogeneity.**
(DOCX)
