# Supplementary material for: Colorful Niches of Phytoplankton Shaped by the Spatial Connectivity in a Large River Ecosystem: A Riverscape Perspective
Source: PLoS One. 2012 Apr 30;7(4):e35891. doi: 10.1371/journal.pone.0035891 (PMC3340396; doi:10.1371/journal.pone.0035891)
Supplement: Table S1 — Hydraulic and landscape characteristics of the 23 main tributaries flowing into the SLR between Cornwall and Île-aux-Coudres. Flow rates were measured on a daily basis during the sampling period (August 8–15, 2006); n/a refers to non-available data. (DOCX) [file pone.0035891.s002.docx]

**Table S1.** Hydraulic and landscape characteristics of the 23 main tributaries flowing into the SLR between Cornwall and Île-aux-Coudres. Flow rates were measured on a daily basis during the sampling period (August 8–15, 2006); n/a refers to non-available data.

| **Tributary** | **PDZ** | **Length**  **(km)** | **Flow**  **rate**  **(m^3^·sec^-1^)** | **Watershed area**  **(km^2^)** | **% of contribution to**  **total PDZ watershed** |
| --- | --- | --- | --- | --- | --- |
| Ottawa | LSL | 1271 | 1984.8 | 146300 | 98.29 |
| Chateauguay | LSL | 100 | 38.5 | 2543 | 1.71 |
| Des Prairies | FR | n/a | 210 | 146000 | 45.63 |
| Milles-Iles | FR | n/a | 1030 | 146000 | 45.63 |
| L’Assomption | FR | 160 | 75.6 | 4220 | 1.32 |
| Richelieu | FR | 171 | 376.3 | 23720 | 7.41 |
| Maskinongé | LSP | 40 | 19.3 | 1105 | 5.23 |
| Yamaska | LSP | 158 | 86.3 | 4784 | 22.64 |
| Saint-François | LSP | 200 | 225.2 | 10230 | 48.42 |
| Du Loup | LSP | 137 | 25.8 | 1610 | 7.62 |
| Nicolet | LSP | 129 | 73.9 | 3398 | 16.08 |
| Saint-Maurice | FE | 563 | 701.9 | 43250 | 63.37 |
| Bécancour | FE | 196 | 60.1 | 2607 | 3.82 |
| Batiscan | FE | 177 | 97.6 | 4690 | 6.87 |
| Sainte-Anne | FE | 123 | 89 | 2694 | 3.95 |
| Portneuf | FE | 30 | 63.3 | 2642 | 3.87 |
| Jacques-Cartier | FE | 161 | 77.2 | 2512 | 3.68 |
| Chaudière | FE | 185 | 132 | 6682 | 9.79 |
| Etchemin | FE | 123 | 34 | 1466 | 2.15 |
| Saint-Charles | FE | 33 | 12.5 | 550 | 0.81 |
| Montmorency | FE | 120 | 36.9 | 1152 | 1.69 |
| Du Sud | ETZ | 42 | 30.8 | 1250 | 53.69 |
| Sainte-Anne-du-Nord | ETZ | 50 | 23.9 | 1078 | 46.31 |
