# Supplementary material for: Colorful Niches of Phytoplankton Shaped by the Spatial Connectivity in a Large River Ecosystem: A Riverscape Perspective
Source: PLoS One. 2012 Apr 30;7(4):e35891. doi: 10.1371/journal.pone.0035891 (PMC3340396; doi:10.1371/journal.pone.0035891)
Supplement: Table S2 — Water mass characteristics, describing their length, area, flow rate, volume, mean depth (Zm), and tributary hydrological index (THI) expressed as the flow rate of tributary/Zm. (DOCX) [file pone.0035891.s003.docx]

**Table S2.** Water mass characteristics, describing their length, area, flow rate, volume, mean depth (Z_m_), and tributary hydrological index (THI) expressed as the flow rate of tributary/Z_m_.

| **Water masse** | **Length** | **Area** | **Flow**  **rate** | **Volume** | **Z_m_** | **THI** |
| --- | --- | --- | --- | --- | --- | --- |
|  | km | km^2^ | m^3^·sec^-1^ | km^3^ | m | m^2^·sec^-1^ |
| Outaouais | 149.8 | **300*** | 716 | 0.19 | 3.20 | 223.75 |
| Chateauguay | 5.6 | 0.43 | 22.42 | 0.0006 | 1.44 | 15.57 |
| Nord-Ouest | 57.9 | 26.87 | 19.16 | 0.06 | 2.26 | 8.48 |
| R-Y | 45.2 | 1.47 | 395.39 | 0.004 | 2.80 | 141.21 |
| R-Y-SF | 26.5 | 0.13 | 544.16 | 0.0003 | 2.30 | 236.59 |
| SF | 26.4 | **68**** | 148.77 | 0.04 | 1.33 | 111.86 |
| Loup-Yam | 26.4 | 12.36** | 8.22 | 0.00000000001 | 1 | 8.22 |
| Nicolet | 31.8 | **19.98**** | 4.66 | 0.001 | 1.01 | 4.61 |
| Maurice | 32.05 | 0.38 | 468.49 | 0.0009 | 2.33 | 201.07 |
| Bécancour | 6.1 | 0.29 | 5.19 | 0.0002 | 0.83 | 6.25 |
| Batis-SA | 33.8 | 5.11 | 68.63 | 0.01 | 1.92 | 35.74 |
| Portneuf-JC | 24.4 | 4.59 | 27.26 | 0.01 | 2.27 | 12.01 |
| Chaud-Etch | 9.7 | 0.08 | 30.61 | 0.0004 | 5.22 | 5.86 |

*Including Lake des Deux Montagnes and the des Prairies and Milles-Iles rivers

**Calculated from the polygon area of the water mass due to the low number of sounding points.
