# Supplementary material for: Colorful Niches of Phytoplankton Shaped by the Spatial Connectivity in a Large River Ecosystem: A Riverscape Perspective
Source: PLoS One. 2012 Apr 30;7(4):e35891. doi: 10.1371/journal.pone.0035891 (PMC3340396; doi:10.1371/journal.pone.0035891)
Supplement: Text S1 — The St. Lawrence River as a representative model of large river ecosystems. (DOCX) [file pone.0035891.s004.docx]

**Text S1: The St. Lawrence River as a representative model of large river ecosystems.**

Great Lakes system is a true representative model of large river ecosystems in the world in terms of drainage area which in turn is directly related to average discharge rate (Fig. S1). It has the 13^th^-largest drainage area in the world, with a total of 1 610 000 km^2^ drained by its tributaries. It constitutes an ideal site for riverscape studies due to its huge physical heterogeneity mostly induced by the drainage network. The structure of the drainage network was the dominant factor in determining physical heterogeneity through the water-mass distribution and the type and amount of injected material on a multikilometer scale.
