# Supplementary material for: Colorful Niches of Phytoplankton Shaped by the Spatial Connectivity in a Large River Ecosystem: A Riverscape Perspective
Source: PLoS One. 2012 Apr 30;7(4):e35891. doi: 10.1371/journal.pone.0035891 (PMC3340396; doi:10.1371/journal.pone.0035891)
Supplement: Text S2 — Bathymetry and morphological characteristics. (DOCX) [file pone.0035891.s005.docx]

**Text S2: Bathymetry and morphological characteristics.**

At the bathymetric and morphological levels, the three fluvial lakes represent 75% of the total river area, and they globally exhibit the lowest mean depths when compared with the other discontinuities (FR, FE, and ETZ). Mean depth is considered to be the most influential morphometric variable in lakes [[1](#_ENREF_1)], being correlated with light and nutrient availability. Therefore, the observed increase of PAR, i.e. in light quantity, in the water column for fluvial lakes correlates with their lower mean depth values, which also favour exchanges between the aquatic environment and the more abundant wetlands along the shores, as documented by [Massicotte and Frenette [2](#_ENREF_2)] for CDOM composition.

Connecting sections such as the FR and FE display totally different behavior along the continuum, exhibiting reduced confluence and drainage densities; a lower PAR availability in the water column, due to their higher mean depth; and a general morphology characterized by a much longer length and smaller width than a typical lotic profile. Altogether, therefore, their capacity to act as a reactor (with a capacity to transform matter [[2](#_ENREF_2)]) is generally reduced compared with fluvial lakes. Therefore, drainage structures, such as tributary-induced water masses, and bathymetric characteristics, such as mean depth, constitute major discontinuity factors along the “continuum,” combining a lower mean depth in general with a large number of tributary-induced water masses, which strongly influence the inherent optical properties in the receiving river and, ultimately, the water color spectrum.

At the eastern end of Ile d’Orléans, the freshwater meets the saltwater and extreme tidal currents develop, generating an intense mixing zone that results in high turbidity and a strong decrease in PAR availability for primary producers. This critical interface for biogeochemical interactions between continental water and the sea is known for its very high biological productivity (refs. in [[3](#_ENREF_3)]). Further details can be found in [Lapierre and Frenette [4](#_ENREF_4)] and [Pommier et al. [5](#_ENREF_5)]. We have demonstrated that the connectivity strongly operates between the freshwater and the estuary of the St. Lawrence River through the downstream transport of phytoplankton from upstream in the ETZ and supporting the primary consumers.

**References**

1. Wetzel RG (2001) Limnology : lake and river ecosystems. San Diego, Calif.: Academic Press. xvi, 1006 p. p.

2. Massicotte P, Frenette JJ (*in press.*) Spatial connectivity in a large river system: resolving the sources and fate of dissolved organic matter. Ecological Applications.

3. Frenette JJ, Demers S, Legendre L, Boule M, Dodson J (1994) Mixing, Stratification and the Fate of Primary Production in an Oligotrophic Multibasin Lake System (Quebec, Canada). Journal of Plankton Research 16: 1095-1115.

4. Lapierre JF, Frenette JJ (2008) Advection of freshwater phytoplankton in the St. Lawrence River estuarine turbidity maximum as revealed by sulfur-stable isotopes. Marine Ecology Progress Series 372: 19-29.

5. Pommier J, Frenette JJ, Glemet H (2010) Relating RNA:DNA ratio in Eurytemora affinis to seston fatty acids in a highly dynamic environment. Marine Ecology-Progress Series 400: 143-154.
