# Supplementary material for: Colorful Niches of Phytoplankton Shaped by the Spatial Connectivity in a Large River Ecosystem: A Riverscape Perspective
Source: PLoS One. 2012 Apr 30;7(4):e35891. doi: 10.1371/journal.pone.0035891 (PMC3340396; doi:10.1371/journal.pone.0035891)
Supplement: Text S3 — St. Lawrence River network characteristics. (DOCX) [file pone.0035891.s006.docx]

**Text S3: St. Lawrence River network characteristics.**

The riverscape characteristics, including the morphological, bathymetric, and hydraulic records for the 23 inflowing tributaries, are presented in Table S1. We calculated an index of tributary impact on water-mass formation called landscape hydrological index (THI) as follows (equation 1) for the reference river section:

|  | $THI\left(m^{2}\cdot\sec^{-1}\right)=\frac{Q_{t}\left(m^{3}\cdot\sec^{-1}\right)}{Z_{m}(m)}$ | (1) |
| --- | --- | --- |

where Q_t_ is the water flow and Z_m_ the mean water depth. The water-mass characteristics describing their length area, flow rate, volume, mean depth (Z_m_), and tributary hydrological impact (THI) appear in Table S2.
