# Supplementary material for: Colorful Niches of Phytoplankton Shaped by the Spatial Connectivity in a Large River Ecosystem: A Riverscape Perspective
Source: PLoS One. 2012 Apr 30;7(4):e35891. doi: 10.1371/journal.pone.0035891 (PMC3340396; doi:10.1371/journal.pone.0035891)
Supplement: Text S4 — Structure of the drainage network and physical heterogeneity. (DOCX) [file pone.0035891.s007.docx]

**Text S4: Structure of the drainage network and physical heterogeneity.**

A total of 23 tributaries (discharge rates > 7 m^3^ s^-1^) flow through a watershed covering 1 600 000 km^2^ (human population of 45 million people), where land use is dominated by a high degree of urbanization near Montreal and by areas of agriculture, pastures, forests, and wetlands in the mid and lower reaches of the stream system [5]. The inflowing tributaries lead to the formation of several parallel water masses with distinct physical and biogeochemical properties [7,16]. Such hydrological heterogeneity is likely to induce pronounced gradients in environmental conditions, with resulting changes in phytoplankton communities.

This study demonstrate that the structure of the drainage network was the dominant factor in determining physical heterogeneity through the water-mass distribution and the type and amount of injected matter. The degree connectivity and thus transfer of material between habitats in the SLR is determined by the spatial structure of the river network and morphology of the riverscape. Among the 23 tributaries, the Ottawa River shows the maximum flow rate, with a value of 1984.8 m^3^ sec^-1^, followed by the Milles-Îles (1030 m^3^ ·sec^-1^), the Saint-Maurice (701.9 m^3^ sec^-1^), the Richelieu (376.3 m^3^ sec^-1^), and the Saint-François (225.2 m^3^ sec ^-1^) rivers. These five rivers also exhibit the highest contribution of drainage area to their respective PDZ (expressed as percent contribution to the PDZ watershed, excluding upstream), with values varying between 98% for the Ottawa River and 48% for the Saint-François River (Table 1A). Moreover, the LSL and the initial portion of the FR (transects 3, 4, and 5) receive an additional contribution of sewage water from the cities of Montréal and Longueuil (see Fig. 1 in main article).

Based on their CDOM and suspended particulate inorganic matter content, the SLR was characterized by 14 major water masses distributed from LSF through the ETZ, inclusive (see Fig. 1 in main article, Table S2). Their relative distribution changed markedly across the PDZs as a result of differences in confluence density and location, as well as the flow rates of tributary inflows, coupled with the variations in the mean depth of the SLR. The interaction of these factors imposed the pattern of water-mass distribution. For instance, the distance from the confluent (length) and volume per unit area (Z_m_) occupied by the water masses in the SLR shows a positive relationship with the discharge rate of the tributaries (r^2^ = 0.27 (p = 0.06) and 0.35 (p < 0.05) respectively; Fig.2A).

After leaving Lake Ontario, only one water mass flows downstream in LSF. Thereafter, a water-mass network develops, with related changes in the number and distribution of water masses within each PDZ. For instance, in LSL the Ottawa River flows into the northern part of the lake and forms a water mass flowing downstream, on the northern part of the FR and, finally, through LSP. This represents the most important water-mass contribution to the riverscape in terms of flow rate, but also in terms of amplitude of distribution, with a 150 km downstream intrusion from the confluence (Table 1A). In LSP, six additional tributaries join the river network: Saint-François, Yamaska, Richelieu, Nicolet, Maskinongé, and du Loup (Fig. 1), for a total of eight water masses, which impose a new hydrodynamic regime to the fluvial lake in terms of residence time and physical and chemical properties (Frenette et al. 2006). These new water masses traveled between 26.4 and 45.2 km further downstream from their confluence (Table S2). At the outlet of LSP, all water masses mix thoroughly as a result of the funnel effect due to the narrowing of the river and the increase in mean depth (Fig. 2). A two-water-mass distribution develops afterward in the FE, with the successive intrusion of six new tributaries along the 150 km distance that separates Trois-Rivières and Québec (see Fig. 1 in main article). For example, 10 km after the LSP outlet, the Saint-Maurice River creates a new water mass, which flows on the north shore along the G-L water mass for 32.05 km and initiates a new two-water-mass regime. Considering its high discharge rate, the Saint-Maurice water mass is, surprisingly, distributed over a small area (0.38 km^2^) in the FE. We attribute this to the increase in the mean depth of the SLR at the confluence area (see Fig. 2 in main article) and to the higher density and lower temperature of the Saint-Maurice, which allows the water mass to sink more deeply. Nevertheless, the volume occupied per unit area is very high (Table S2). The two-water-mass distribution pattern continues until Québec City, with gradual mixing and turnover (replacement) by new inflowing tributaries from the north (5) and the south (2) shores. These water masses traveled between 15.8 km and 98.5 km alongside the central (G-L) water mass until Québec City, where they mixed thoroughly, again creating (as upstream in LSF), a unique water-mass regime that lasted until the ETZ. Furthermore, water masses with the highest LHI occupied the largest volume per unit area in the SLR and showed the highest flow rates (Table S2; see Fig. 1 in the main article), emphasizing the key role of tributary discharge and receiving-channel bathymetry in controlling water-mass intrusion and distribution in the SLR. The degree of connectivity and thus transfer of material between habitats in the St. Lawrence is thus determined by the spatial structure of the river and morphology of the riverscape.

**References**

1. Wetzel RG (2001) Limnology : lake and river ecosystems. San Diego, Calif.: Academic Press. xvi, 1006 p. p.

2. Massicotte P, Frenette JJ (*in press.*) Spatial connectivity in a large river system: resolving the sources and fate of dissolved organic matter. Ecological Applications.

3. Frenette JJ, Demers S, Legendre L, Boule M, Dodson J (1994) Mixing, Stratification and the Fate of Primary Production in an Oligotrophic Multibasin Lake System (Quebec, Canada). Journal of Plankton Research 16: 1095-1115.

4. Lapierre JF, Frenette JJ (2008) Advection of freshwater phytoplankton in the St. Lawrence River estuarine turbidity maximum as revealed by sulfur-stable isotopes. Marine Ecology Progress Series 372: 19-29.

5. Pommier J, Frenette JJ, Glemet H (2010) Relating RNA:DNA ratio in Eurytemora affinis to seston fatty acids in a highly dynamic environment. Marine Ecology-Progress Series 400: 143-154.
